# Genetic deletion of microsomal prostaglandin E synthase-1 promotes imiquimod-induced psoriasis in mice

**DOI:** 10.1186/s41232-025-00385-2

**Published:** 2025-06-06

**Authors:** Fumiaki Kojima, Yuka Hioki, Miori Sumida, Yoshiko Iizuka, Hitoshi Kashiwagi, Kei Eto, Shiho Arichi, Shotaro Maehana, Makoto Kubo, Haruhito A. Uchida, Takafumi Ichikawa

**Affiliations:** 1 Department of Pharmacology, Kitasato University School of Allied Health Sciences, 1-15-1 Kitasato, Minami-Ku, Sagamihara, 252-0373 Japan; 2 Department of Regulation Biochemistry, Kitasato University Graduate School of Medical Sciences, 1-15-1 Kitasato, Minami-Ku, Sagamihara, 252-0373 Japan; 3 Regenerative Medicine and Cell Design Research Facility, Kitasato University School of Allied Health Science, 1-15-1 Kitasato, Minami-Ku, Sagamihara, 252-0373 Japan; 4 Department of Public Health, Kitasato University Graduate School of Medical Sciences, 1-15-1 Kitasato, Minami-Ku, Sagamihara, 252-0373 Japan; 5 Faculty of Pharmaceutical Sciences, Hokkaido University, Kita 12-Jo, Nishi 6-Chome, Kita-Ku, Sapporo, 060-0812 Japan; 6 Department of Physiology, Kitasato University School of Allied Health Sciences, 1-15-1 Kitasato, Minami-Ku, Sagamihara, 252-0373 Japan; 7 Department of Environmental Microbiology, Kitasato University Graduate School of Medical Sciences, 1-15-1 Kitasato, Minami-Ku, Sagamihara, 252-0373 Japan; 8 Department of Chronic Kidney Disease and Cardiovascular Disease, Faculty of Medicine, Dentistry, and Pharmaceutical Science, Okayama University, 2-5-1 Shikata-Cho, Kita-Ku, Okayama, 700-8558 Japan

**Keywords:** Psoriasis, Inflammation, Immunity, Interleukin-17 A, Cyclooxygenase, Prostaglandin E synthase, Prostaglandin E_2_, γδ T cell

## Abstract

**Background:**

Psoriasis is a chronic inflammatory disease associated with abnormalities in the immune system. Microsomal prostaglandin E synthase-1 (mPGES-1), a terminal enzyme for prostaglandin (PG) E_2_ biosynthesis, is highly expressed in the skin of psoriasis patients. However, the detailed role of mPGES-1 in psoriasis remains unclear. In the present study, we aimed to investigate the role of mPGES-1 in psoriasis-like skin inflammation induced by imiquimod (IMQ), a well-established model of psoriasis.

**Methods:**

Psoriasis was induced in mPGES-1-deficient (mPGES-1^−/−^) and wild-type (WT) mice by administering IMQ for 6 days. Psoriasis was evaluated based on the scores of the macroscopic symptoms, including skin scaling, thickness, and redness, and on the histological features. The skin expression of mPGES-1 was determined by real-time polymerase chain reaction and Western blotting. The impact of mPGES-1 deficiency on T-cell immunity was determined by flow cytometry and γδ T-cell depletion in vivo with anti-T-cell receptor (TCR) γδ antibody.

**Results:**

The inflamed skin of mPGES-1^−/−^ mice showed severe symptoms after the administration of IMQ. Histological analysis further showed significant exacerbation of psoriasis in mPGES-1^−/−^ mice. In WT mice, the mPGES-1 expression was highly induced at both mRNA and protein levels in the skin, and PGE_2_ increased significantly after IMQ administration, while the PGE_2_ production was largely abolished in mPGES-1^−/−^ mice. These data indicate that mPGES-1 is the main enzyme responsible for PGE_2_ production in the skin. Furthermore, the lack of mPGES-1 increased the numbers of IL-17A-producing γδ T cells in the skin with IMQ-induced psoriasis, and γδ T-cell depletion resulted in a reduction of the facilitated psoriasis symptoms under the condition of mPGES-1 deficiency.

**Conclusions:**

Our study results demonstrate that mPGES-1 is the main enzyme responsible for skin PGE_2_ production, and that mPGES-1 deficiency facilitates the development of psoriasis by affecting the development of T-cell-mediated immunity. Therefore, mPGES-1 might impact both skin inflammation and T-cell-mediated immunity associated with psoriasis.

**Supplementary Information:**

The online version contains supplementary material available at 10.1186/s41232-025-00385-2.

## Introduction

Psoriasis is a chronic inflammatory skin disease characterized by skin thickening, scaling, and erythema all over the body. The characteristic skin rash on the entire body places a heavy physical and emotional burden on patients, which could lead to the deterioration of their quality of life (QOL) [1]. Psoriasis is believed to be related to immune abnormalities and is characterized by T-cell-mediated hyperproliferation of keratinocytes and abundant T-cell accumulation at the site of inflammation. Triggered by environmental and genetic factors, including stress and external stimuli, interleukin (IL)−23 produced by the activated dendritic cells induces several subsets of T cells, and IL-17 A produced by these T cells activates the keratinocytes. The production of tumor necrosis factor-alpha (TNFα), interferon-gamma (IFNγ), and IL-8 is promoted around this IL-23/IL-17 A system, which is thought to cause a chain reaction of psoriasis exacerbations [2, 3]. In recent clinical practice, biologics have been used against cytokines, such as IL-17 A and TNFα, which are considered exacerbating factors and are important in the pathogenesis and progression of this disease, and they have shown some efficacy [4]. However, no curative treatment based on the pathogenesis of the disease has yet been established, and it is desirable to elucidate the details of the mechanism of the pathogenesis and to establish new treatment methods based on these mechanisms.

Prostaglandin (PG) E_2_ is an unsaturated fatty acid with a 20-carbon chain that plays various roles in pathophysiology and homeostasis. Its production is initiated by the release of arachidonic acid from phospholipids, constituting cell membranes, by the action of phospholipase A_2_. The subsequent process is regulated by a further sequential enzymatic pathway involving cyclooxygenase (COX) and PGE synthase (PGES) [5, 6]. COX has the following two isozymes: COX-1 and COX-2. COX-1 is thought to be a constitutive enzyme because of its homeostatic expression in various tissues throughout the body. Contrarily, COX-2 is well known to have an inducible property, and its expression is induced by a variety of inflammatory stimuli. On the basis of the results of early studies, COX-1 and COX-2 had been considered the key step in PGE_2_ synthesis, but subsequent studies have discovered that the PGES isozymes are responsible for the final step of PGE_2_ synthesis downstream from COXs. To date, at least three distinct PGES isozymes, i.e., cytosolic PGES (cPGES), microsomal PGES-1 (mPGES-1), and mPGES-2, have been identified, and the properties and roles of these enzymes are being intensively studied [7–10].

PGE_2_ exerts a wide variety of physiological effects by binding to four different specific receptor subtypes (EP_1_, EP_2_, EP_3_, and EP_4_), which mediate different cellular signaling [11, 12]. Among the EP receptor subtypes, EP_2_ and EP_4_ play a role in regulating the pathological events in an IL-23-induced psoriasis model [13]. An evaluation of biopsy samples from patients with psoriasis has shown that both COX-2 expression and EP_4_ receptor increase in the psoriatic lesions of the skin. In fact, some patients with psoriasis often use nonsteroidal anti-inflammatory drugs (NSAIDs), which inhibit COX activity, for treating joint disease including psoriatic arthritis without considering the possibility that these drugs could worsen the skin inflammation of psoriasis. Notably, many previous reports have implicated the worsening of psoriasis with the initiation of NSAIDs [14–22]. Conversely, a clinical study has also found benefits in terms of psoriasis improvement when NSAIDs are used [23]. Thus, the impact of NSAIDs on psoriasis is quite variable, and the whole concept of the role of arachidonic acid metabolites including PGE_2_ in psoriasis has not been fully clarified yet.

mPGES-1 is an inducible enzyme that acts downstream of COXs and specifically catalyzes the conversion of PGH_2_ to PGE_2_ [7, 8]. Several studies in mPGES-1-deficient (mPGES-1^−/−^) mice have provided novel findings on the role of mPGES-1 as a key mediator of many physiological and pathophysiological events in various disease states associated with immune responses and inflammation [24–30]. We have previously reported that mPGES-1-driven PGE_2_ regulates T-cell-mediated antigen-specific humoral responses in vivo, suggesting an important role of mPGES-1 and the mPGES-1-driven PGE_2_ in the development of acquired immune response [31, 32]. mPGES-1 also regulates the antigen-specific Th17 and Th1 responses in an autocrine and paracrine manner [33]. We have also recently reported that the mPGES-1 deficiency facilitates the development of experimental colitis by affecting the development of colonic T-cell-mediated immunity [34, 35]. These previous findings strongly suggested the pivotal roles of mPGES-1 in pathogenic T-cell immunity.

The imiquimod (IMQ)-induced psoriasis model is widely used as a well-established model of psoriasis [36]. IMQ is an agonist of toll-like receptor (TLR) 7 and TLR8, which are mainly expressed on dendritic cells and macrophages. IMQ stimulates the production of cytokines, including TNFα, IL-12, and IL-23, from dendritic cells and macrophages by binding to TLR7, and it also causes activated T-cell subsets to produce IL-17 A and IFNγ, which in turn stimulate cellular immunity. These pathological mechanisms of the IMQ-induced psoriasis model are considered quite similar to those of human psoriasis [37, 38]. It has been demonstrated that mPGES-1 is highly expressed in the skin of psoriasis patients [13, 39], suggesting the importance of mPGES-1 in the pathogenesis of psoriasis. However, the significance and role of the overexpression of mPGES-1 in the pathogenesis of psoriasis remain largely unknown. In the present study, we aimed to investigate the role of mPGES-1 in the development of psoriasis using an IMQ-induced psoriasis model to elucidate the pathogenesis of this disease.

## Materials and methods

### Mice

The generation of mPGES-1^−/−^ mice has been reported by Uematsu et al. [28]. mPGES-1^−/−^ and wild-type (WT) control mice have the genetic background of Balb/c strain generated by backcrossing at least 10 generations to Balb/c mice [35]. The mice were housed in cages placed in a specific pathogen-free barrier facility and were cared for and handled in accordance with the guidelines of the Animal Research and Ethics Committee of Kitasato University and the Safety Committee for Recombinant DNA Experiments of Kitasato University. All animal experiments were approved by the Animal Research and Ethics Committee of Kitasato University (approval number: Ei-ken 24–06), and all experiments involving the mPGES-1^−/−^ mice were approved by the Safety Committee for Recombinant DNA Experiments of Kitasato University (approval number: 4929).

### IMQ-induced psoriasis

Male mice aged between 8 and 12 weeks were used in the present study. Their backs were shaved, and they received a daily topical application of 62.5 mg of Beselna Cream (Mochida Pharmaceutical, Tokyo, Japan) containing 5% IMQ, which is known to present symptoms similar to those seen in human psoriasis. For the control animals, 62.5 mg of Vaseline (Fujifilm Wako Pure Chemical, Osaka, Japan) was administered.

### Scoring the severity of inflammation

The severity of skin inflammation was evaluated using the clinical psoriasis area and severity index, except that for the mouse model, as the affected skin area was not taken into account in the scoring. Specifically, it was assessed by scoring the following main symptoms of psoriasis: redness, scaling, and thickness of the skin of each mouse. The thickness of the skin on the back was measured using calipers (Mitutoyo, Kawasaki, Japan). Each parameter was given a score between 0 and 4 (0, normal; 1, slight; 2, moderate; 3, marked; and 4, very marked). The weight changes of the mice while administering IMQ were also measured.

### Histological assessment of psoriasis

On day 6 after the start of exposure to IMQ, the mice were euthanized under anesthesia, and skin samples were collected. The samples were fixed in 4% paraformaldehyde and then embedded in paraffin. The sections with 3.5-μm thickness were stained with hematoxylin and eosin (H&E). The epidermal thickness was measured with NIS-Elements D3.00 microscope imaging software (Nikon Instruments Inc., Tokyo, Japan).

### Cell isolation

Isolation of the cells from the skin was performed according to the modified method described by Lou et al. [40]. In brief, two pieces of the skin with a size of 1 × 1 cm (50–100 mg) were collected from the back of mice euthanized under anesthesia, and each piece was cut into small pieces. The pieces were treated in DMEM/high glucose (Sigma-Aldrich, St. Louis, MO, USA) containing a 5 mg/mL Dispase-II Solution (Roche Diagnostics, Rotkreuz, Switzerland) at 37 °C for 1 h. After washing, the samples were further incubated with a dermis dissociation buffer containing 0.1 mg/mL DNase I (Roche Diagnostics) and 1 mg/mL Collagenase P (Roche Diagnostics) in DMEM/high glucose at 37 °C for 1 h with 5% CO_2_. The enzymatic reaction was then stopped with DMEM/high glucose containing 10% fetal bovine serum, and the suspension was filtered through a 40-μm cell strainer to collect the skin cells. Splenocytes were also isolated, as described elsewhere [31].

### Flow cytometry (FCM) analysis

The cells were incubated with an anti-CD16/32 antibody (TruStain FcX; BioLegend) to block the FcγII/III receptor-mediated nonspecific antibody binding before the surface staining of the cell surface markers. The cells were then stained with fluorochrome-conjugated anti-mouse monoclonal antibodies (BioLegend) against CD45 (clone: 30-F11), CD3 (clone: 17 A2), T-cell receptor (TCR)β (clone: H57-597), or TCRγδ (clone: GL3). The cells were also stained with antibodies against TCRβ or TCRγδ before intracellular staining for IFNγ and IL-17 A. For the analysis of Vγ chain of γδ Tcells, cells were stained with antibodies against TCRγδ and Vγ4 chain (clone: UC3-10 A6) before intracellular staining for IL-17 A. Isotype controls were also used to characterize the background signal from off-target antibody binding. The Zombie Aqua Fixable Viability Kit (BioLegend) was used in all analyses to remove dead cells and avoid background or unspecific staining of dead cells. For the staining of IL-17 A- and IFNγ-producing T cells, intracellular staining for IFNγ (clone: XMG1.2; Biolegend) and IL-17 A (clone: TC11-18H10.1; Biolegend) was performed after the stimulation of cells, staining of surface molecules, and fixation and permeabilization of cells. Briefly, single-cell suspensions were incubated with the leukocyte activation cocktail with BD GolgiPlug (BD Biosciences, Franklin Lakes, NJ) for 4 h in vitro in an RPMI 1640 medium supplemented with 10% fetal bovine serum, penicillin/streptomycin, and freshly added 50 μmol/L 2-mercaptoethanol. The Cytofix/Cytoperm Plus Fixation/Permeabilization kit (BD PharMingen, Franklin Lakes, NJ, USA) was used to fix, permeabilize, and stain the cells per manufacturer’s instructions. The gating strategy was always performed in the following order: total events — lymphocyte gate (FSC-A/SSC-A) — living cells (alive/dead), with subsequent gating indicated in each experiment.

### Myeloperoxidase (MPO) assay

The MPO activity was measured to quantify the inflammatory cell infiltrates in the skin, according to a previous report [41]. In brief, skin tissue samples were homogenized and sonicated in 50-mM potassium phosphate buffer (pH 6.5) containing 0.5% (w/v) hexadecyl trimethylammonium bromide. The sonicated samples were subjected to centrifugation at 1000 g for 10 min at 4 °C. The supernatant (10 µL) was transferred to a 96-well plate containing 100 µL of reaction buffer [50-mM potassium phosphate buffer (pH 6.0), 0.16 mg/mL o-dianosidine (Sigma-Aldrich), and 0.0005% hydrogen peroxide]. A commercially available MPO (Calbiochem, San Diego, CA, USA) was used as the standard. The absorbance at 450 nm was measured with a Benchmark Microplate Reader (BioRad, Hercules, CA, USA) after a 5-min incubation.

### Real-time polymerase chain reaction (PCR) analysis

Total RNA was isolated from the skin using a NucleoSpin RNA kit (Macherey–Nagel, Duren, Germany). First-strand cDNAs were synthesized with SuperScript VILO (Thermo Fisher Scientific, Waltham, MA, USA); then, real-time PCR was performed with a Thunderbird SYBR qPCR Mix (Toyobo, Osaka, Japan) in the ABI 7500 Real-Time PCR System (Thermo Fisher Scientific). The information on the primer sets (Eurofins, Luxembourg City, Luxembourg) used in the present study is described in our previous study [34]. The cycling conditions of the PCR reaction were as follows: 1 min at 95 °C, followed by 40 cycles of 15 s each at 95 °C and 1 min at 60 °C. The threshold cycle value was normalized with reference to glyceraldehyde 3-phosphate dehydrogenase.

### Western blot analysis

The tissues were homogenized and lysed in a buffer containing 40 mmol/L Tris/HCl (pH 7.4), 150 mmol/L NaCl, 2 mmol/L EDTA, 1 mmol/L dithiothreitol, 1% Triton X-100, 2 mmol/L sodium orthovanadate, 10 mmol/L NaF, and 10 mmol/L sodium pyrophosphate supplemented with a protease inhibitor cocktail mixture (Sigma, St. Louis, MO, USA). The protein contents were measured using a BCA protein assay kit (Thermo Fisher Scientific), and bovine serum albumin was used as a standard. The samples were separated by sodium dodecyl sulfate–polyacrylamide gel electrophoresis, and then the proteins were transferred onto a PVDF membrane (GE HealthCare, Little Chalfont, UK). After a blocking procedure, the membrane was incubated with anti-mPGES-1 (No. 160140; Cayman Chemicals, Ann Arbor, MI, USA), anti-cPGES (No. 160150; Cayman Chemicals), anti-COX-2 (No. 160106; Cayman Chemicals), anti-COX-1 (No. 160109; Cayman Chemicals), anti-hematopoietic PGD synthase (hPGDS, No. 10004348; Cayman Chemicals), or anti-*β*-actin (A5441; Sigma) antibody and then incubated with a secondary antibody coupled to horseradish peroxidase (Jackson ImmunoResearch Laboratories, PA, USA). After washing, the protein was detected by enhanced chemiluminescence (GE HealthCare).

### Measurement of PGE_2_ and PGD_2_

The tissues were homogenized in 70% methanol supplemented with 30-μM indomethacin. The homogenates were centrifuged at 15,000 g at 4 °C for 20 min. The supernatant was evaporated under a nitrogen gas stream and suspended in an enzyme immunoassay buffer, and the levels of PGE_2_ and PGD_2_ as a MOX-PGD_2_ (a stable metabolite of PGD_2_) were measured by using enzyme-linked immunosorbent assay kits (Cayman Chemicals), according to the manufacturer’s protocol [42]. Optical density was measured with the Benchmark Microplate Reader (BioRad).

### In vivo* γδ T-cell depletion*

Mice were treated with 0.2 mg of anti-TCRγδ polyclonal antibody (clone: UC7-13D5; Bio X Cell, West Lebanon, NH) or 0.2 mg of isotype-matched control antibody (clone: polyclonal Armenian hamster IgG; Bio X Cell) via intraperitoneal injections on day 1 relative to the start of the IMQ administration. The depletion of TCRγδ-positive T cells was confirmed through an FCM analysis of the T-cell population in the skin by staining with fluorochrome-conjugated anti-mouse monoclonal antibodies (BioLegend) against TCRγδ (clone GL3) and TCRβ (clone H57-597).

### In vivo* CD4-positive T-cell depletion*

Mice were treated with 0.1 mg of anti-CD4 monoclonal antibody (clone GK1.5; Bio X Cell, West Lebanon, NH) or 0.1 mg of isotype-matched control antibody (clone LTF-2; Bio X Cell) via intraperitoneal injections on day 1 relative to the start of IMQ administration [43]. Depletion of CD4-positive T cells was confirmed by FCM analysis of T-cell populations in the peripheral blood and spleen by staining with fluorochrome-conjugated anti-mouse monoclonal antibodies (BioLegend) against CD3 (clone 17 A2), CD4 (clone RM4.4), and CD8 (clone 53–6.7).

### Statistical analysis

Data are expressed as the means ± SEM. Statistical analysis was performed with SigmaSat 3.5 software (Systat Software, Inc., San Jose, CA, USA). Data from more than two groups were compared through an analysis of variance (ANOVA) followed by Tukey’s multiple comparison test, and data from two groups were compared through a *t*-test after testing for normal distribution. *P* < 0.05 was considered statistically significant.

## Results

### Exacerbation of IMQ-induced psoriasis in mice with mPGES-1 genetic deletion

**Fig. 1:**
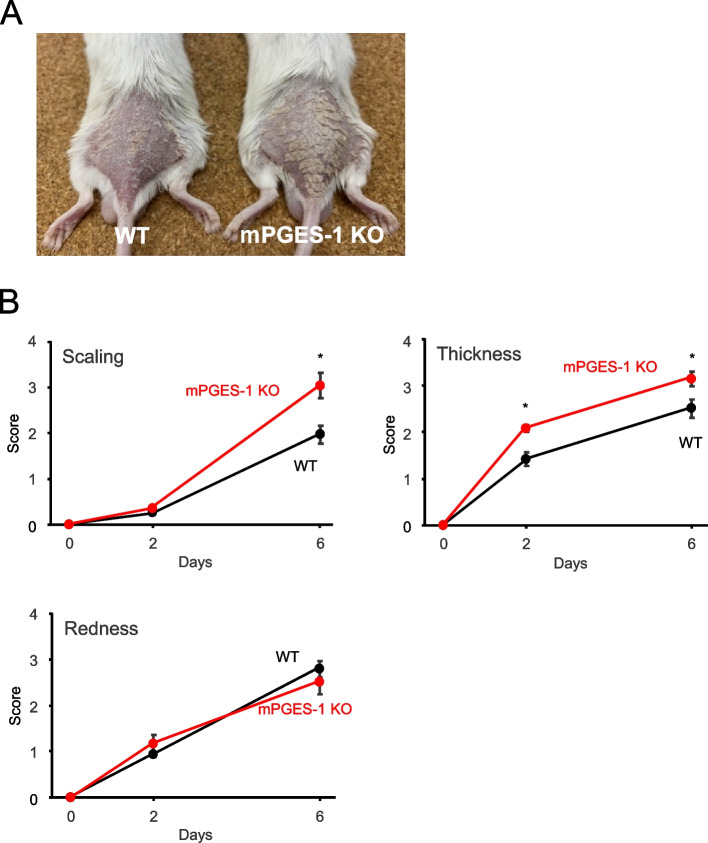
Clinical course of IMQ-induced psoriasis in mice with mPGES-1 genetic deletion. **A** Typical examples of WT and mPGES-1^−/−^ mice with psoriasis-like pathology induced by IMQ treatment for 6 days. **B** Time course of change in the scaling, skin thickness, and redness scores of WT and mPGES-1^−/−^ mice after the indicated days of exposure to IMQ (*n* = 14 to 16). *P < 0.05; ANOVA followed by Tukey’s multiple comparison test

The typical examples of WT and mPGES-1^−/−^ mice with psoriasis-like pathology induced by IMQ treatment for 6 days are shown in Fig. 1. The pathological evaluation of the IMQ-induced psoriasis pathology model was performed by scoring the samples according to the evaluation criteria. The scores for skin scaling and thickness were significantly higher in the mPGES-1^−/−^ mice than in the WT mice, indicating an exacerbation of the psoriasis pathology (Fig. 1). Contrarily, there was no difference in the score of skin redness, which is one of the characteristic symptoms of psoriasis, between the mPGES-1^−/−^ and WT mice. Both the mPGES-1^−/−^ and WT mice showed mild weight loss after IMQ treatment, but no difference was observed between the two groups.

### Histological analysis of the skin samples from the IMQ-induced psoriasis pathological model

**Fig. 2:**
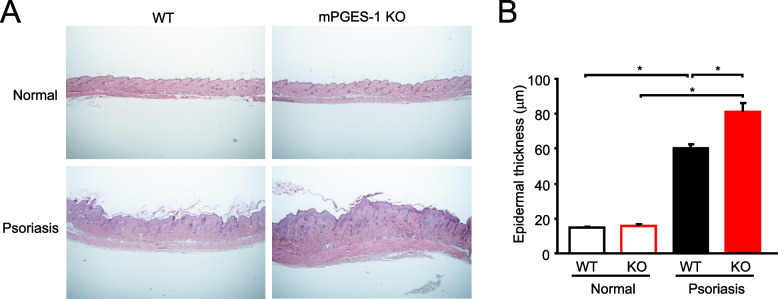
Histological analysis of IMQ-induced psoriasis in mPGES-1^−/−^ mice. **A** Skin samples of WT and mPGES-1^−/−^ mice were collected on day 6 after the start of exposure to IMQ, and sections were stained with H&E. The results are representative samples of skin tissues from the WT and mPGES-1^−/−^ mice. **B** Epidermal thickness was assessed on H&E-stained sections (*n* = 8 to 11). *P < 0.05; ANOVA followed by Tukey’s multiple comparison test

The stratum corneum and stratum spinosum of the epidermis are known to be markedly thickened in the skin of patients with psoriasis due to the abnormal proliferation of epidermal cells. Therefore, epidermal thickness was measured under a microscope as an indicator of disease progression in an IMQ-induced psoriasis pathological model (Fig. 2). The epidermis and hair follicles were stained with hematoxylin, whereas the dermis and muscle layer were stained with eosin. The microscopic measurements of the epidermal thickness were not different between the WT and mPGES-1^−/−^ mice. Six days of IMQ treatment resulted in a marked epidermal thickening in WT mice with an induced psoriasis pathology, as compared to the normal mice. The IMQ-induced skin thickening was significantly enhanced in the mPGES-1^−/−^ mice than in the WT mice. Histological analysis also revealed that the psoriasis pathology was exacerbated in the mPGES-1^−/−^ mice.

### Inflammatory cell infiltration and MPO activity in the skin for pathological evaluation

**Fig. 3:**
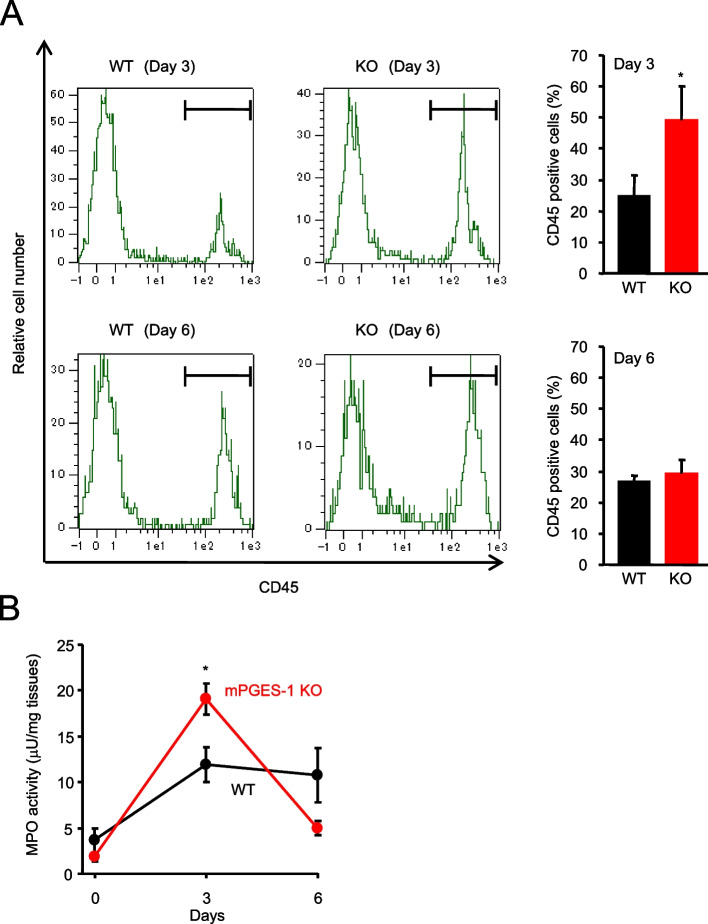
Effect of mPGES-1 gene deletion on inflammatory cell infiltration and MPO activity in IMQ-induced psoriasis. **A** Representative FCM histogram showing leukocytes stained with an anti-CD45 antibody in the skin isolated from WT and mPGES-1^−/−^ mice with psoriasis at days 3 and 6 after the IMQ treatment. The ratio of CD45-positive cells in the skin on days 3 and 6 after the start of exposure to IMQ in both genotypes (*n* = 3). *P < 0.05 vs WT; *t*-test. **B** The MPO activity in the skin samples from the WT and mPGES-1^−/−^ mice treated with IMQ for the indicated days was measured to quantify the inflammatory cell infiltrates in the skin (*n* = 5 to 12). *P < 0.05; ANOVA followed by Tukey’s multiple comparison test

The infiltration of inflammatory cells in the skin of the IMQ-induced psoriasis pathological model was investigated. The results of flow cytometry analysis of skin cells from WT and mPGES-1^−/−^ mice treated with IMQ for 3 and 6 days after isolation and leukocyte staining with anti-CD45 antibody are shown in Fig. 3A. The percentage of CD45-positive cells was significantly increased in the skin of the mPGES-1^−/−^ mice treated with IMQ for 3 days as compared with the WT mice. Contrarily, the percentage of CD45-positive cells was similar in the mPGES-1^−/−^ and WT mice treated with IMQ for 6 days.

Additionally, the MPO activity was measured as an indicator of neutrophil infiltration in the skin (Fig. 3B). The results showed a marked increase in MPO activity in the skin of mPGES-1^−/−^ mice treated with IMQ for 3 days, correlating with the increase in the percentage of CD45-positive cells shown in Fig. 3A. These results suggest that the lack of mPGES-1 may exacerbate the symptoms of psoriasis in mPGES-1^−/−^ mice in terms of inflammatory cell infiltration in the skin, suggesting that the presence of mPGES-1 may be protective against the pathology of psoriasis.

### mRNA expression of PGE_2_ biosynthetic enzymes in IMQ-induced psoriasis

**Fig. 4:**
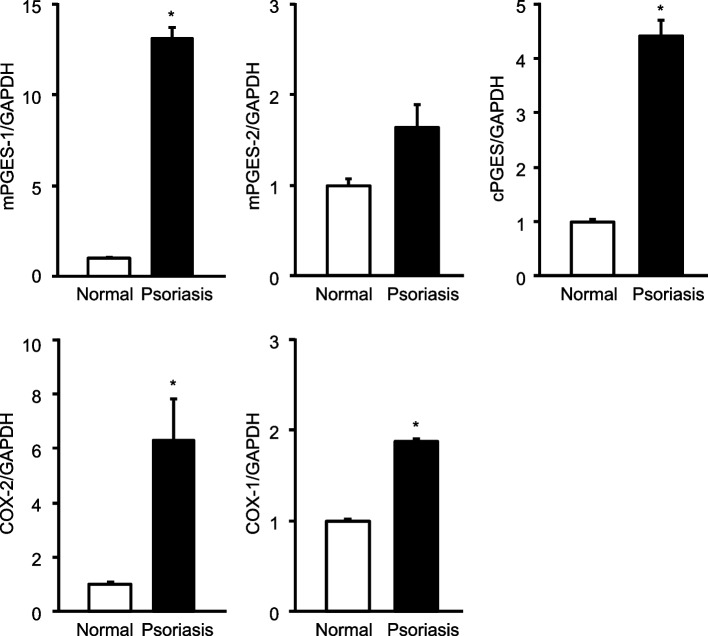
The expressions of mRNA for the PGE_2_ biosynthetic enzymes in the skin after exposure to IMQ. The expression of mRNA for PGES and COX isozymes in the skin samples obtained from the WT mice treated or not treated with IMQ for 6 days was analyzed by real-time PCR (*n* = 3 to 6). The levels of mRNA expression are shown as the fold induction relative to the expression in WT mice without treatment with IMQ (assigned the value “1”). *P < 0.05 vs normal; *t*-test

The mRNA expression of PGE_2_ biosynthetic enzymes in the skin of WT mice at 6 days after the induction of psoriasis with the administration of IMQ was analyzed by real-time PCR (Fig. 4). The mRNA expression was significantly increased for mPGES-1 and cPGES among the three PGE isozymes. Furthermore, the COX isozymes, which act upstream of the PGES, showed significant increases in mRNA expression for both COX-1 and COX-2.

### Protein expression of prostanoid biosynthetic enzymes and prostanoid production in the skin

**Fig. 5:**
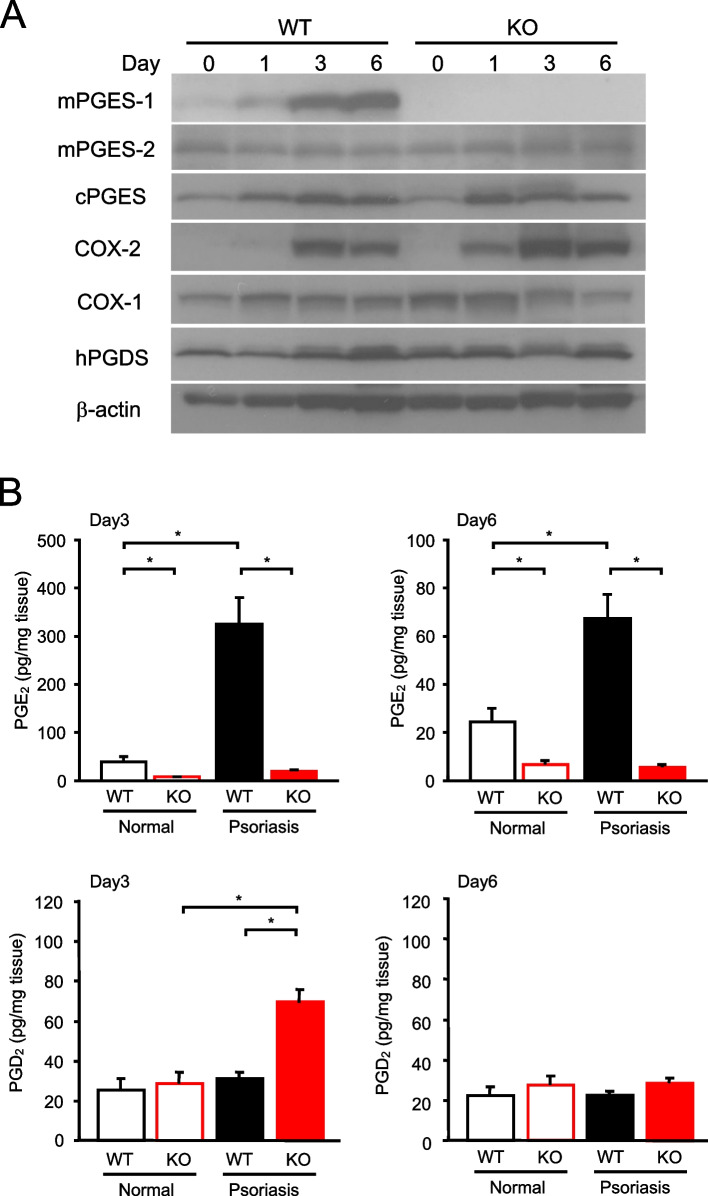
mPGES-1 protein expression and prostanoid production in the skin with psoriasis induced by the administration of IMQ. **A** The expression of protein for PGES and COX isozymes in the skin samples obtained from mice treated with IMQ for the indicated days was examined by Western blot analysis. The results are representative blots for the WT and mPGES-1^−/−^ mice. **B** The PGE_2_ and PGD_2_ levels in the skin from the mice treated or not treated with IMQ for 3 and 6 days were measured by ELISA (*n* = 5 to 11). *P < 0.05; ANOVA followed by Tukey’s multiple comparison test

We next analyzed the time course of the change in the protein expression of PGE_2_ biosynthetic enzymes in the skin (Fig. 5A). The protein expression of mPGES-1 was induced in the skin of WT mice according to the number of days of IMQ treatment, with the highest increase observed on day 6. Contrarily, mPGES-1 was not expressed in the skin of the mPGES-1^−/−^ mice. In both the WT and mPGES-1^−/−^ mice, cPGES was upregulated on day 1 after the IMQ administration, and its expression was maintained at least until day 6. The mPGES-2 expression was not affected by the IMQ administration or mPGES-1 deficiency. The expression of COX-2, an upstream enzyme, was strongly induced on day 3 in both the WT and mPGES-1^−/−^ mice and showed a decreasing trend by day 6. Interestingly, the induction of COX-2 was greater in the mPGES-1^−/−^ mice than in the WT mice. Contrarily, the COX-1 expression tended to slightly increase on the first day of IMQ treatment, but it decreased during the IMQ treatment period; this trend was more markedly observed in the mPGES-1^−/−^ mice than in the WT mice. The protein expression of hPGDS, an isozyme of PGD synthase for PGD_2_ biosynthesis, was not changed by IMQ administration or mPGES-1 deficiency.

We next determined the role of mPGES-1 in skin prostanoid production under normal and psoriatic skin conditions (Fig. 5B). On day 3 after the induction of psoriasis, the PGE_2_ level increased significantly in the WT mice, but not in the mPGES-1^−/−^ mice. Notably, even in normal skin without treatment with IMQ, the genetic deletion of mPGES-1 resulted in a greater reduction of skin PGE_2_, as compared with WT mice. Similar results were also observed in the skin of WT and mPGES-1^−/−^ mice on day 6 after the induction of psoriasis, even though the PGE_2_ level was lower on day 6 than on day 3. These data clearly indicate that mPGES-1 is the main synthase responsible for PGE_2_ production not only in psoriatic skin but also in healthy skin.

By inhibiting most of the PGE_2_ production in the mPGES-1^−/−^ mice, we speculated that the intermediate substance, PGH_2_, may shunt to the other prostanoid. Therefore, we examined the PGD_2_ production in the skin of mice treated with IMQ for 3 and 6 days to induce psoriasis. The skin of mPGES-1^−/−^ mice on day 3 after the induction of psoriasis showed increased PGD_2_ production as compared with that of WT mice. Thus, it was suggested that a portion of PGH_2_ may shunt to PGD_2_ under conditions wherein the PGE_2_ production is strongly suppressed by mPGES-1 deficiency. However, no difference in the amount of PGD_2_ production was observed between the skin of the WT and mPGES-1^−/−^ mice on day 6 after psoriasis induction.

### Facilitating psoriasis-related cytokine expression in mPGES-1-deficient mice

**Fig. 6:**
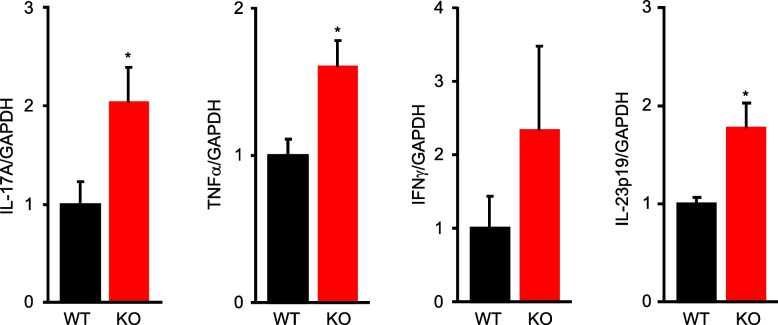
The expression of cytokines in the skin of mPGES-1^−/−^ mice with IMQ-induced psoriasis. The expression of mRNA for IL-17 A, TNFα, IFNγ, and IL-23p19 in the skin samples obtained from mice treated with IMQ for 6 days was analyzed by real-time PCR (*n* = 4 to 6). The levels of mRNA expression are shown as the fold induction relative to that of the WT mice (assigned the value “1”). *P < 0.05 vs WT mice; *t*-test

Immune abnormalities are largely involved in the pathogenesis and progression of psoriasis, and the production and action of a variety of cytokines contribute to the pathogenesis and progression of the disease. Thus, we aimed to analyze the mRNA expression of major cytokines, including IL-17 A, TNFα, and IFNγ in the skin of the IMQ-induced psoriasis pathological model (Fig. 6). The skin of mPGES-1^−/−^ mice treated with IMQ for 6 days showed increased mRNA expressions of IL-17 A and TNFα as compared to those of WT mice, although the IFNγ expression was not significantly different between the two groups. We also examined the expression level of IL-23p19, a component of IL-23, which was shown to be a critical cytokine for the development of pathogenic Th17 cells as well as IL-17 A-producing γδ T cells [44]. The expression of IL-23p19 was significantly higher in mPGES-1^−**/−**^ mice than in WT mice. These results correlated well with the pathophysiological and histopathological evidence of psoriasis observed under mPGES-1 deficiency in the present study.

### Changes in the T-cell population in mPGES-1^−/−^ mice with IMQ-induced psoriasis

**Fig. 7:**
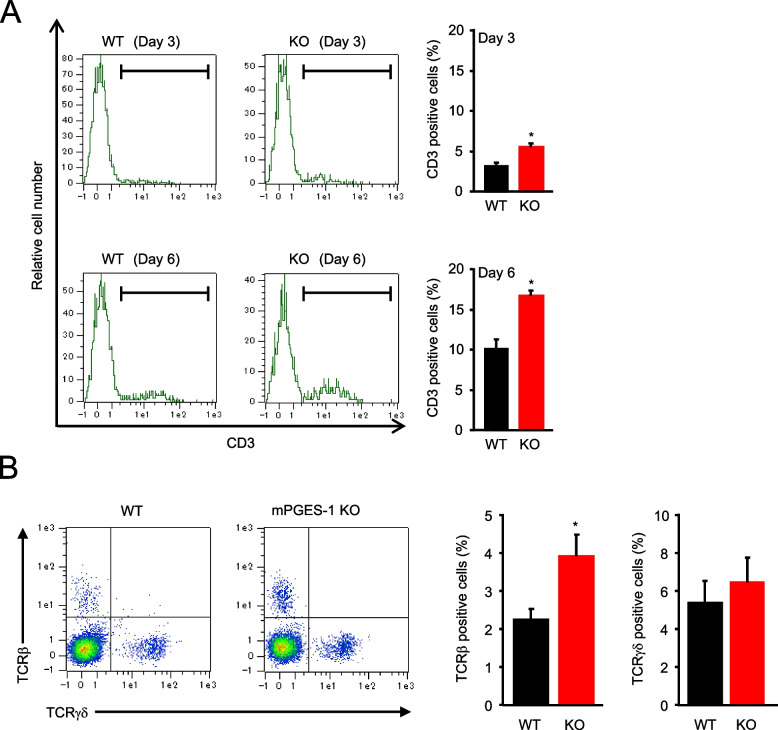
Profile of T-cell subsets in the skin of mPGES-1^−/−^ mice with IMQ-induced psoriasis. **A** Representative FCM histogram showing CD3-positive T cells in the skin isolated from the WT and mPGES-1^−/−^ mice with psoriasis at days 3 and 6 after the IMQ treatment. The ratio of CD3-positive cells in the skin on days 3 and 6 after the start of exposure to IMQ in both genotypes (*n* = 3). **B** Representative FCM plot of TCRβ- and TCRγδ-positive T cells in the skin isolated from the WT and mPGES-1^−/−^ mice with psoriasis on day 6. The ratio of αβ T and γδ T cells in the skin on day 6 after the start of exposure to IMQ (*n* = 7 to 9). *P < 0.05 vs WT; *t*-test

The skin cells were isolated from the mice with psoriasis induced by 3 and 6 days of IMQ treatment, and the percentage of T cells in the skin was analyzed using an anti-CD3 antibody (Fig. 7A). The results showed that the percentage of CD3-positive T cells was significantly increased in the mPGES-1^−/−^ mice, as compared to that in WT mice, on both days 3 and 6 after the induction of psoriasis.

To further analyze the skin on day 6 after psoriasis induction, we analyzed the αβ T cells and γδ T cells based on the differences in TCR expression (Fig. 7B). The percentage of αβ T cells detected as TCRβ-positive was significantly increased in the skin of mPGES-1^−/−^ mice, as compared with that of WT mice. Contrarily, the percentage of γδ T cells detected as TCRγδ -positive did not differ between the WT and mPGES-1^−/−^ mice.

### Genetic deletion of mPGES-1 results in enhanced generation of IL-17 A-producing γδ T cells

**Fig. 8:**
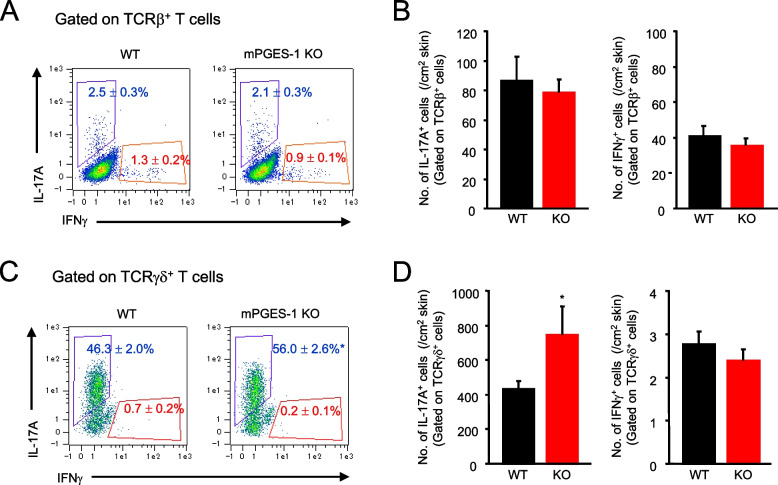
Generation of IL-17 A- and IFNγ-producing T-cell subsets in the skin of mPGES-1^−/−^ mice with IMQ-induced psoriasis. **A** Representative FCM plot and ratio of IL-17 A- and IFNγ-producing αβ T cells in the skin isolated from the WT and mPGES-1^−/−^ mice with psoriasis on day 6. **B** The number of IL-17 A- and IFNγ-producing αβ T cells per unit area of the skin on day 6 after the start of exposure to IMQ (*n* = 5). **C** Representative FCM plot and ratio of IL-17 A- and IFNγ-producing γδ T cells in the skin isolated from the WT and mPGES-1^−/−^ mice with psoriasis on day 6. **D** The number of IL-17 A- and IFNγ-producing γδ T cells per unit area of the skin on day 6 after the start of exposure to IMQ (*n* = 5). *P < 0.05 vs WT; *t*-test

To further determine the impact of mPGES-1 on the developing T-cell immunologic responses associated with psoriasis, after inducing psoriasis, we determined the fraction of αβ T cells and γδ T cells producing IL-17 A and IFNγ in the skin cells of mPGES-1^−**/−**^ and WT mice. The skin cells were isolated from the mice with psoriasis induced by IMQ treatment for 6 days, stimulated ex vivo, surface stained for TCRβ or TCRγδ, fixed, stained for intracellular IL-17 A and IFNγ, and analyzed by FCM. The results showed that for IL-17 A-producing αβ T cells gated on the TCRβ -positive T cells, no difference in both the percentage and number of cells per unit skin area was observed between the mPGES-1^−/−^ and WT mice (Fig. 8A and B). Contrarily, both the percentage of IL-17 A-producing γδ T cells and the number of cells per skin unit area were significantly increased in the skin of mPGES-1^−/−^ mice, as compared with the WT mice (Fig. 8C and D). In both the IFNγ-producing αβ T and IFNγ-producing γδ T cells, no difference was observed between the mPGES-1^−/−^ and WT mice.

**Fig. 9:**
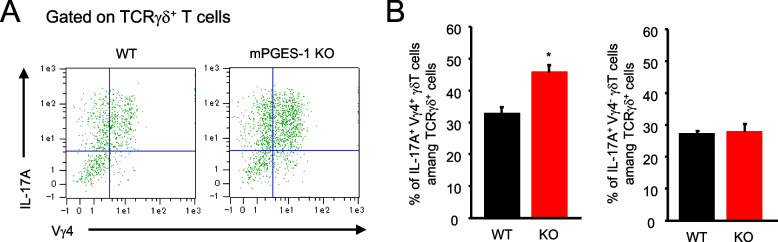
Effect of mPGES-1 gene deletion on the population of Vγ4-positive/negative IL-17 A-producing γδ T cells in IMQ-induced psoriasis. **A** Representative FCM plot of Vγ4-positive/negative IL-17 A-producing γδ T cells in the skin isolated from the WT and mPGES-1^−/−^ mice with psoriasis on day 6. **B** The ratio of Vγ4-positive IL-17 A-producing T cells and Vγ4-negative IL-17 A-producing T cells among γδ T cells in the skin on day 6 after the start of exposure to IMQ (*n* = 4). *P < 0.05 vs WT; *t*-test

We also investigated the usage of Vγ chains of γδ T cells in the skin of WT and mPGES-1^−/−^ mice. The percentage of Vγ4-positive IL-17 A-producing T cells among γδ T cells increased in the skin of mPGES-1^−/−^ mice than in that of WT mice (Fig. 9A and B). Notably, the percentage of Vγ4-negative IL-17 A-producing γδ T cells was similar in both genotypes, suggesting the major participation of Vγ4-positive T cells in IL-17 A-producing γδ T cells.

### Attenuated symptoms of IMQ-induced psoriasis by neutralizing the antibodies for γδ T-cell depletion in mPGES-1^−/−^ mice

**Fig. 10:**
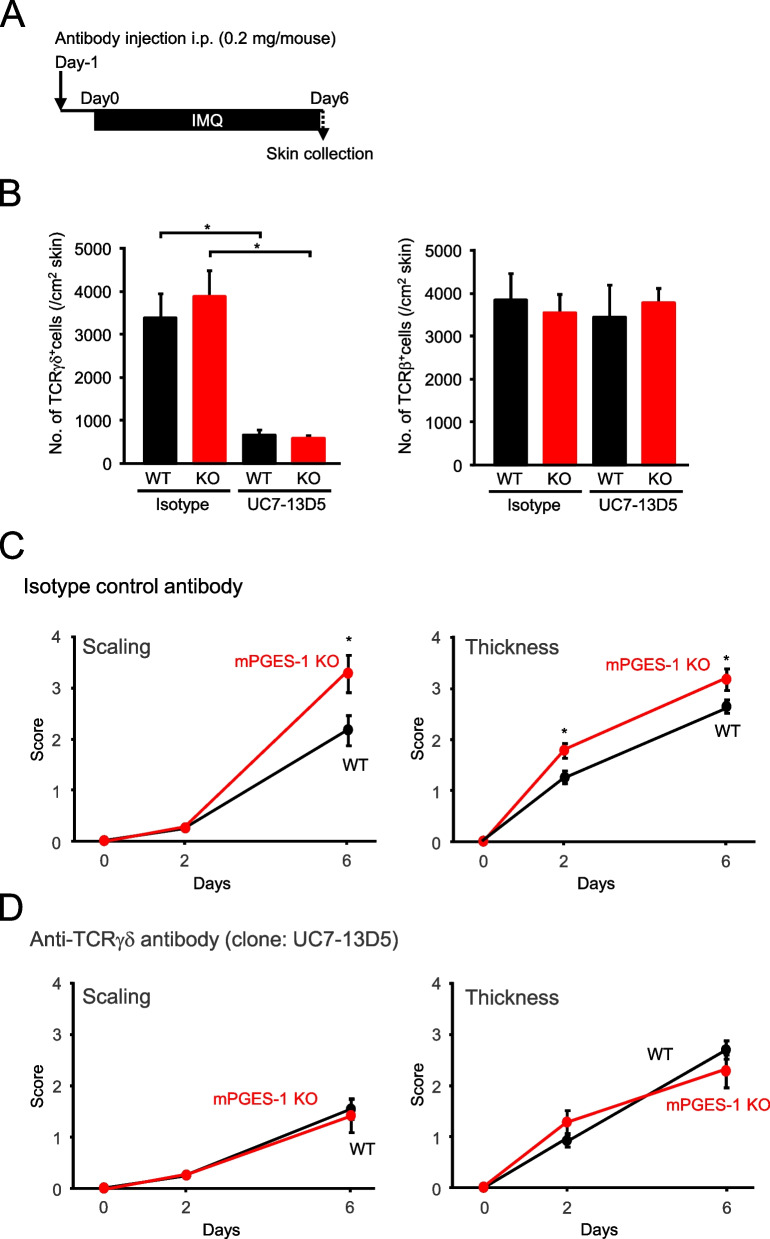
Effect of γδ T-cell depletion on the exacerbated IMQ-induced psoriasis in mPGES-1^−/−^ mice. **A** Schematic representation of the experimental plan. **B** The efficacy of in vivo γδ T-cell depletion was confirmed by flow cytometry analysis of the T-cell population in the skin. **C**, **D** Effect of isotype control (**C**) and anti-TCR γδ (**D**) antibodies on the scaling and skin thickness scores of the WT and mPGES-1^−/−^ mice after indicated days of exposure to IMQ (*n* = 9 to 15). *P < 0.05; ANOVA followed by Tukey’s multiple comparison test

To investigate the functional role of T cells in the exacerbated IMQ-induced psoriasis under the condition of mPGES-1 deficiency, the course of IMQ-induced psoriasis in mPGES-1^−/−^ mice was studied upon γδ T-cell depletion by treatment with an anti-TCRγδ polyclonal antibody (clone UC7-13D5) prior to the administration of IMQ (Fig. 10A). The efficacy of γδ T-cell depletion was confirmed via an FCM analysis of the T-cell population in the skin (Figs. 10B and S1). The treatment with UC7-13D5 effectively reduced the number of γδ T cells in vivo, whereas the isotype control antibody had no effect. Both UC7-13D5 and isotype control did not affect the number of αβ T cells in the skin.

We evaluated the pathogenesis of IMQ-induced psoriasis under these conditions with reduced γδ T cells. The mPGES-1^−/−^ mice treated with isotype control antibodies showed a marked exacerbation of skin scaling and thickness as compared with the WT mice (Fig. 10C), but the difference was largely abolished under the condition of γδ T-cell depletion by treatment with an anti-TCR γδ antibody in vivo (Fig. 10D). The abovementioned data strongly suggest that γδ T cells may play an important role in the exacerbation of the psoriasis pathology observed in mPGES-1^−/−^ mice.

**Fig. 11:**
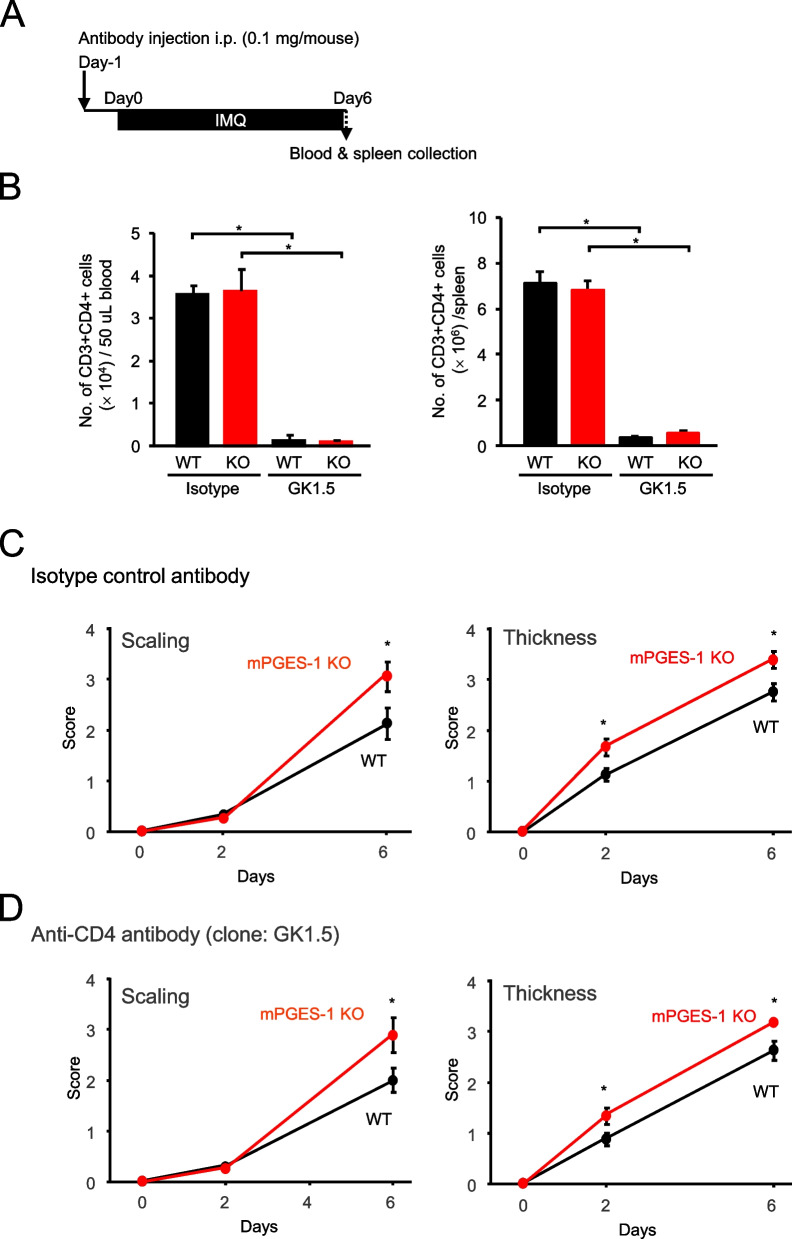
Effect of CD4-positive T-cell depletion on the exacerbated IMQ-induced psoriasis in mPGES-1^−/−^ mice. **A** Schematic representation of the experimental plan. **B** The efficacy of in vivo CD4-positive T-cell depletion was confirmed by flow cytometry analysis of T-cell population in the peripheral blood and spleen. **C**, **D** Effect of isotype control antibody (**C**) and anti-CD4 antibody (**D**) on scaling and skin thickness score of WT and mPGES-1^−/−^ mice after indicated days of exposure to IMQ (*n* = 10 to 13). *P < 0.05; ANOVA followed by Tukey multiple comparison test

We also checked the effect of anti-CD4 monoclonal antibody (clone GK1.5) that depletes CD4-positive Th cells including αβ T cells but not γδ T cells (Fig. 11A). The efficacy of CD4-positive T-cell depletion was confirmed by FCM analysis of the T cell population (Fig. 11B and S2). As shown in Fig. 10B, the treatment with GK-1.5 effectively reduced the number of CD3^+^CD4^+^ T cells in vivo, whereas the isotype control antibody had no effect (Figs. 11B and S2). Both GK1.5 and the isotype control antibody did not affect the population of CD3^+^CD8^+^ T cells (Fig. S2). Evaluation of skin scaling and thickening also showed no effect of anti-CD4 antibody treatment, and the IMQ-induced psoriasis symptoms were still significantly exacerbated in mPGES-1^−/−^ mice compared to WT mice (Fig. 11C and D).

## Discussion

The present study utilizing an IMQ-induced psoriasis model, which is known to exhibit a pathology very similar to that of human psoriasis, found for the first time that the psoriasis pathology in mPGES-1^−/−^ mice is significantly exacerbated as compared with that in WT mice. This finding suggests that mPGES-1 may have a protective property against the pathogenesis of psoriasis. Furthermore, in the psoriatic regional skin of mPGES-1^−/−^ mice, there is a rapid and abundant accumulation of CD45-positive leukocytes on day 3 after psoriasis induction, correlating with the high MPO activity, an indicator of neutrophil infiltration. These results suggest that mPGES-1 may have protective effects against inflammation at a relatively early stage of the pathogenesis of psoriasis, which is characterized by a chronic course of repeated exacerbations and remissions.

The present study also found that mPGES-1 expression was markedly induced in the skin of WT mice, along with increased COX-2 expression, during the course of psoriasis induced by the administration of IMQ. Several studies have previously reported that the expression of COX-2 is increased in the skin of the IMQ-induced psoriasis model [45, 46]; however, research focusing on PGES isozymes in an IMQ-induced psoriasis model has not been conducted. Interestingly, the lack of mPGES-1 enhanced the induction of COX-2 in response to IMQ in the skin. This result is also consistent with the findings of previous studies utilizing mPGES-1^−/−^ mice with colitis induced by dextran sodium sulfate and trinitrobenzene sulfonic acid [34, 35]. These results indicate that mPGES-1 and its derived PGE_2_ may regulate the expression of their upstream enzymes in a feedback loop. Additionally, the difference in the time-course pattern between the mPGES-1 and COX-2 protein expressions in response to IMQ is among the important findings of the present study. Similarly, a previous study has reported on the difference in the time-course profile of the mPGES-1 and COX-2 expressions in the immune tissues, including the spleen and lymph nodes, of a type II collagen-induced arthritis (CIA) mouse model [32]. The different time-course profiles of the mPGES-1 and COX-2 expressions are also consistent with the findings of in vitro studies using a variety of cell types and inflammatory stimuli [29, 47]. The varying transcriptional regulation of mPGES-1 and COX-2 may help to explain the different time-course profiles of the expression of these enzymes [48–50]. Furthermore, we demonstrated that the induction of mPGES-1 is critical for the marked increase of PGE_2_ production in the skin during the course of psoriasis. The time-course pattern of PGE_2_ production, with higher levels observed on day 3 than on day 6, appeared to be correlated with the time course of COX-2 expression rather than with that of mPGES-1 expression, suggesting the importance of COX-2 as a late-limiting enzyme for the PGE_2_ production in the skin during the course of psoriasis.

We detected an increased production of PGD_2_ at a relatively early stage of the pathogenesis of psoriasis in the skin of mPGES-1^−/−^ mice, which seemed to be due to the shunting of PG precursors down the PGD_2_ synthetic pathway in the absence of mPGES-1. We also detected an enhanced colonic COX-2 expression as a result of mPGES-1 deletion. These results suggest that an elevation of COX-2 expression and the resultant increase in the availability of PGH_2_ as the common substrate for the generation of prostanoid accounts for the increased PGD_2_ levels observed in mPGES-1^−/−^ mice. These results are further supported by the findings of our previous in vitro [29, 51–53] and in vivo [34, 35] studies under the condition of mPGES-1 deficiency, which showed shunting toward PGD_2_ production. Given that PGD_2_ production was increased in the skin of mPGES-1^−/−^ mice after the induction of psoriasis, we confirmed the effect of PGD_2_ in IMQ-induced psoriasis pathology under the condition of mPGES-1 deficiency using antagonists specific for each PGD_2_ receptor (DP) subtype DP_1_ and DP_2_. Our results showed no significant effect of the pharmacological inhibition of the PGD_2_ receptors DP_1_ and DP_2_ on the facilitated symptoms of psoriasis observed in the IMQ-treated mPGES-1^−/−^ mice (Fig. S3). These results suggest that PGD_2_, which is increased in the skin of psoriasis-induced mPGES-1^−/−^ mice, is unlikely to exacerbate or alleviate the facilitated symptoms of IMQ-induced psoriasis under the condition of mPGES-1 deficiency. A previous study using mice with the C57BL/6 NCrSlc strain showed that in the ear treated with IMQ, the level of thromboxane (TX) B_2_, a stable metabolite of TXA_2_, significantly increased among the prostanoid, whereas the level of PGE_2_ decreased [54]. This significant reduction of PGE_2_ level in the IMQ-treated ear is in contrast to the findings of our current study, which showed a significant increase of PGE_2_ level in the IMQ-treated skin on the back of Balb/c mice. A previous study has suggested that the pathogenesis of IMQ-induced psoriasis is controlled by different mechanisms, depending on the site treated with IMQ (ear versus skin on the back) [55]. Another report also documented the differences in the clinical symptoms of psoriasis induced by IMQ depending on the genetic background of mice [56]. Therefore, the disparate results obtained from the two studies may be explained by the fact that those authors topically applied IMQ on the ear of C57BL/6 NCrSlc mice, which was in contrast to our experiments, wherein we induced psoriasis on the skin at the back of the Balb/c mice.

It is noteworthy that the psoriasis-induced mPGES-1^−/−^ mice had increased splenic weight as compared to the WT mice. Moreover, the results of the histological analysis showed that the area occupied by the white splenic cord increased in the mPGES-1^−/−^ mice, as compared with the WT mice (data not shown). Since the spleen is among the major secondary lymphoid tissues and a key organ in the immune response, our observations imply that the systemic immune system may be hyperactive in response to IMQ in the mPGES-1^−/−^ mice. However, the present study could not elucidate the details of the immunological role of mPGES-1 in the spleen. Alternatively, our present study focused on the immune system of the skin, the primary site of psoriasis, to clarify the role of mPGES-1 in the T-cell-mediated immune response in psoriasis. We demonstrated for the first time that the lack of mPGES-1 facilitates the expression of IL-17 A and TNFα in the skin in the mice with IMQ-induced psoriasis. The IMQ treatment reportedly induces IL-17 A and TNFα expressions in the skin; these cytokines are essential for the development of psoriasis [2, 57]. In addition, in the skin of psoriasis-induced mPGES-1^−/−^ mice, we found an increased percentage of CD3-positive T cells as compared to that of WT mice, with a marked increase in the percentage of αβ T cells, although the percentage of γδ T cells in the skin was not significantly different between the mPGES-1^−/−^ and WT mice. Among the T cells, helper T (Th) 17 cells are well known to be the major Th cell subset producing IL-17 A and belong to αβ T cells, but γδ T cells, which are relatively abundant in the skin, are also the major IL-17 A-producing cells during psoriasis [58–60]. Surprisingly, we found that the number of IL-17 A-producing γδ T cells was significantly increased in the skin of mPGES-1^−/−^ mice compared with that of WT mice, whereas the number of IL-17 A-producing αβ T cells including Th17 cells in the skin of mPGES-1^−/−^ mice was comparable with that of WT mice. Furthermore, the population of Vγ4-positive IL-17 A-producing T cells among γδ T cells was higher in the skin of mPGES-1^−/−^ mice compared with that of WT mice, while the population of Vγ4-negative IL-17 A-producing γδ T cells was similar in both genotypes. These findings suggest that the IL-17 A-producing Vγ4-positive γδ T cells may be involved in the exacerbation of IMQ-induced psoriasis under the condition of mPGES-1 deficiency. Furthermore, we also found that the depletion of γδ T cells strongly suppressed skin scaling and thickening, which are the primary symptoms of IMQ-induced psoriasis in mPGES-1^−/−^ mice, to the same degree as those seen in WT mice. These results strongly suggest that γδ T cells could be involved in the exacerbation of psoriasis in mPGES-1^−/−^ mice.

Since IL-23 is critical for the development of pathogenic IL-17 A-producing γδ T cells [44], we examined the expression of IL-23p19, a component of IL-23, in the skin of mPGES-1^−/−^ mice and WT mice during IMQ-induced psoriasis. We found that the expression of IL-23p19 is enhanced in the skin of mPGES-1^−/−^ mice compared to that of WT mice, which may explain why IL-17 A-producing γδ T cells increase in the skin of mPGES-1^−/−^ mice. A study using T-cell receptor δ^−/−^ mice clearly demonstrated that IL-23-responsive dermal γδ T cells are the major IL-17 producers in the skin of IMQ-induced psoriasis as well as patients with psoriasis [61]. IL-23p19^−/−^ mice and IL-17 A^−/−^ mice show complete suppression of the symptoms caused by IMQ, respectively [2]. Further molecular analysis focused on specific cell types, such as γδ T cells and IL-23-producing dendritic cells, remains to be studied to elucidate the detailed mechanisms by which mPGES-1 deficiency affects the differentiation and activation of γδ T cells.

Lee et al. clearly demonstrated that PGE_2_ promotes IL-17 production from Th17 cells, thereby inducing inflammation in psoriasis in the IL-23 injection model of psoriasis [13]. In fact, the study indicated that injected IL-23-stimulated Th17 cells produce PGE_2_, which acts on the EP_2_ and EP_4_ in the Th17 cell itself in an autocrine manner with enhanced expression of the IL-23 receptor on Th17 cells, while this study did not focus on the IL-17 A-producing γδ T cells. IL-23 is a key cytokine produced by antigen-presenting cells such as dendritic cells that is necessary for the development of pathogenic IL-17 A-producing T cells including both Th17 cells and γδ T cells [36]. It is remarkable that the IL-23 injection model of psoriasis exerts limited inflammation due to the activation of only a single pathway induced by exogenous IL-23. On the other hand, the IMQ-induced psoriasis model has the advantages of producing more dramatic inflammation that is complexly associated with a variety of endogenous mediators and various types of activated cells including antigen-presenting cells such as dendritic cells. IL-23 is endogenously produced by dendritic cells and drives IL-17 A-dependent skin inflammation in the IMQ-induced psoriasis model [2, 62]. In the present study using IMQ-induced psoriasis, IL-17 A-producing γδ T cells were increased in the skin of mPGES-1^−/−^ mice, while no differences in IL-17 A-producing αβ T cells including Th17 were observed between the mPGES-1^−/−^ and WT mice. A recent study using NF-κB^−/−^ mice showed that IL-17 A-producing Vγ4-/Vδ4-positive γδ T cells, but not CD4-positive traditional Th17, are major cellular sources of IL-17 A associated with the pathophysiology of IMQ-induced psoriasis [63]. Thus, these disparate results obtained from our present study and a report by Lee et al. [13] could be explained by the fact that those authors performed their studies using the IL-23 injection model of psoriasis, in contrast to our experiments performed with the IMQ-induced psoriasis model.

The functions of PGE_2_ in IL-17 A-producing T cells including Th17 cells as well as γδ T cells have been reported in both in vitro and in vivo models. PGE_2_ is shown to promote the expansion of Th17 cells [64]. We and colleagues also reported that antigen-experienced mPGES-1^−/−^ CD4^+^ T cells produced significantly less IL-17 A than their WT CD4^+^ T-cell counterparts upon restimulation with their specific antigens, suggesting the importance of Th17 cells in mPGES-1/PGE_2_-mediated immune response [65]. We also previously reported that mPGES-1 deficiency results in a reduction in the severity of CIA [32]. PGE_2_ exacerbates arthritis development in the CIA through the inflammatory IL-23/IL-17 pathway [66]. Of interest, the majority of γδ T cells, especially Vγ4/Vδ4-positive γδ T cells, produce IL-17 A in CIA [67]. IL-23^−/−^ mice showed a significant reduction of IL-17 A-producing γδ T cells, suggesting that IL-23 is critical for IL-17 A production in γδ T cells [68]. While some studies reported that PGE_2_ regulates T cells in a facilitative manner in the immune system, our recent studies showed that mPGES-1 deficiency promotes the development of experimental colitis, mouse models of inflammatory bowel disease, by affecting IL-17 A-producing CD4^+^ T cells, suggesting a protective role for mPGES-1 and its derived PGE_2_ by negatively regulating immune responses involving Th17 cells [34, 35]. In the present study using IMQ-induced psoriasis, IL-17 A-producing γδ T cells were increased in the skin of mPGES-1^−/−^ mice. Furthermore, depletion of γδ T cells, but not depletion of CD4-positive T cells including Th17 cells, suppressed symptoms of IMQ-induced psoriasis in mPGES-1^−/−^ mice. The effects of mPGES-1/PGE_2_ on the T-cell immune system, particularly IL-17-producing T cells, may be diverse in various pathological conditions and experimental systems, and further analysis using comprehensive analysis such as single-cell RNA sequencing is needed to elucidate the details of the direct and possibly other cell-mediated indirect mechanisms of action of the mPGES-1/PGE_2_ system on the T-cell-mediated immune responses.

To date, there is no animal model that accurately replicates all the characteristics of human psoriasis. Among the mouse models of psoriasis, the IMQ-induced psoriasis model closely resembles the human psoriasis in terms of the epidermis hyperplasia and inflammatory infiltrate, which is comprised of various cells from the immune system, including T cells, neutrophils, and dendritic cells [36]. The alteration of numerous gene expressions by IMQ was also consistent with the characteristics of human psoriasis. IMQ also altered gene expression in a manner consistent with human psoriasis. Nevertheless, this model does not perfectly mirror the pathology of human psoriasis in all its complexities [69]. In particular, the IMQ model represents an acute inflammatory condition, whereas human psoriasis is characterized by chronic inflammation. In consideration of its acute and severe characteristics in disease progression in the IMQ-induced psoriasis model, the findings with the IMQ model may be partly restricted to being directly applicable to the chronic pathology of human psoriasis. To date, several mouse models of psoriasis have been described that mimic a variety of features of psoriasis [70]. Thus, future studies using multiple models might be needed to clarify the further detailed role of mPGES-1 and its derived PGE_2_ in relation to human psoriasis with chronic inflammation.

The present study clearly demonstrated that mPGES-1 is the main PGES responsible for PGE_2_ production in the skin, and that mPGES-1-associated PGE_2_ plays a protective role in psoriasis, partly by regulating the immune system associated with γδ T cells. The IL-17 A-producing γδ T cells in the skin may be a possible representative that could be used to explain the mechanism of facilitated psoriasis in the absence of mPGES-1. mPGES-1 is a promising candidate for drug development because the inhibition of mPGES-1 could specifically decrease the elevated PGE_2_ level associated with various autoimmune inflammatory diseases. However, in psoriasis, the protective effect mediated by the mPGES-1-driven PGE_2_ appears to be indispensable for preventing the hyperactivation of the pathogenic T-cell immune response and resultant skin inflammation. Thus, the present study also provides potentially important information on the possible disadvantages of pharmacological mPGES-1 inhibition in patients with psoriasis. To investigate the therapeutic efficacy and safety of mPGES-1 inhibitors, future studies need to assess whether mPGES-1 inhibitors can mimic the results observed in mPGES-1^−/−^ mice and examine how their effects differ from those of traditional COX inhibitors.

While the current findings suggest that mPGES-1 may play a protective role in psoriasis, it is essential to consider how these results can inform clinical treatment strategies. As the development of mPGES-1 inhibitors progresses, it is crucial to evaluate the potential risks associated with their use, particularly regarding the modulation of T-cell-mediated immunity. Future studies should focus on elucidating the mechanisms by which mPGES-1 influences immune responses and inflammation in the skin. This understanding will be vital for determining the therapeutic implications of targeting mPGES-1 in psoriasis management, ensuring that treatment approaches balance efficacy with the preservation of immune function.

## Conclusions

mPGES-1 is the main enzyme responsible for the PGE_2_ production in the skin, and mPGES-1 deficiency facilitates the development of psoriasis by affecting the development of T-cell-mediated immunity. Therefore, mPGES-1 might impact both skin inflammation and T-cell-mediated immunity associated with psoriasis.

## Supplementary Information

Supplementary Material 1. Figure S1. In vivo depletion of γδ T cells by anti-TCRγδ polyclonal antibody. Schematic representation of the experimental plan. Solid allows indicate timepointat which the intraperitoneal injection of the antibody were performed. The efficacy of γδ T-cell depletion was confirmed through an FCM analysis of the skin samples. The administration of anti-TCRγδ antibody was successful in maintaining a significant decrease in the TCRγδ-positive γδ T cells but not TCRβ-positive αβ T cells for 7 days after the antibody administration. Figure S2. In vivo depletion of CD4-positive T cells by anti-CD4 monoclonal antibody. Schematic representation of the experimental plan. Solid allows indicate time-pointat which intraperitoneal injection of the antibody were performed. The efficacy of CD4-positive T cell depletion was confirmed by FCM analysis of T cell population in the peripheral bloodand spleen. FCM analysis confirmed that anti-CD4 antibody treatment effectively reduced the number of CD3^+^ CD4^+^ T cells but not CD3^+^ CD8^+^ T cells in the peripheral blood and spleen of the treated mice compared to the control mice. *P < 0.05 vs. control; *t*-test. Figure S3. Effect of pharmacological DP receptor inhibition on facilitated psoriasis under the condition of mPGES-1 deficiency. Given that PGD_2_ production was increased in the skin of mPGES-1^−/−^ mice after the induction of psoriasis, we investigated the effects of PGD_2_ in the IMQ-induced psoriasis pathology under the condition of mPGES-1 deficiency using antagonists specific for each DP subtype DP_1_ and DP_2_. DP_1_ antagonist BWA868C was administered intraperitoneally to the mPGES-1^−/−^ mice at a dose of 1 mg/kg daily for 6 days immediately before the IMQ treatment, and the psoriasis pathology was evaluated. There was no significant difference in the skin scaling and thickening scores between mPGES-1^−/−^ mice treated with a DP_1_ antagonist and mPGES-1^−/−^ mice treated with a vehicle. The DP_2_ antagonist CAY10471 was administered intraperitoneally to the mPGES-1^−/−^ mice at a dose of 2 mg/kg daily for 6 days immediately before the IMQ treatment. Similarly, there was no significant difference in both scores between the DP_2_ antagonist and vehicle control in mPGES-1^−/−^ mice. These results suggest that PGD_2_, which is increased in the skin of psoriasis-induced mPGES-1^−/−^ mice, is unlikely to exacerbate or alleviate the major symptoms of IMQ-induced psoriasis. *P < 0.05; ANOVA followed by Tukey’s multiple comparison test.

## Data Availability

Data generated or analyzed during this study are included in this published article and its supplementary information files.

## Abbreviations

IL: Interleukin
TNFα: Tumor necrosis factor-α
IFNγ: Interferon-γ
PG: Prostaglandin
COX: Cyclooxygenase
PGES: PGE synthase
cPGES: Cytosolic PGES
mPGES-1: Microsomal PGES-1
mPGES-1^−/−^ mice: Mice lacking mPGES-1
Th: T helper
IMQ: Imiquimod
TLR: Toll-like receptor
WT: Wild-type
H&E: Hematoxylin and eosin
FCM: Flow cytometry
MPO: Myeloperoxidase
PCR: Polymerase chain reaction
GAPDH: Glyceraldehyde 3-phosphate dehydrogenase
hPGDS: Hematopoietic PGD synthase
ANOVA: Analysis of variance
TX: Thromboxane
