# Supplementary material for: Genetic deletion of microsomal prostaglandin E synthase-1 promotes imiquimod-induced psoriasis in mice
Source: Inflamm Regen. 2025 Jun 6;45:18. doi: 10.1186/s41232-025-00385-2 (PMC12142878; doi:10.1186/s41232-025-00385-2)
Supplement: Supplementary file 1 — Supplementary Material 1. Figure S1. In vivo depletion of γδ T cells by anti-TCRγδ polyclonal antibody. Schematic representation of the experimental plan. Solid allows indicate timepointat which the intraperitoneal injection of the antibody were performed. The efficacy of γδ T-cell depletion was confirmed through an FCM analysis of the skin samples. The administration of anti-TCRγδ antibody was successful in maintaining a significant decrease in the TCRγδ-positive γδ T cells but not TCRβ-positive αβ T cells for 7 days after the antibody administration. Figure S2. In vivo depletion of CD4-positive T cells by anti-CD4 monoclonal antibody. Schematic representation of the experimental plan. Solid allows indicate time-pointat which intraperitoneal injection of the antibody were performed. The efficacy of CD4-positive T cell depletion was confirmed by FCM analysis of T cell population in the peripheral bloodand spleen. FCM analysis confirmed that anti-CD4 antibody treatment effectively reduced the number of CD3+ CD4+ T cells but not CD3+ CD8+ T cells in the peripheral blood and spleen of the treated mice compared to the control mice. *P < 0.05 vs. control; t-test. Figure S3. Effect of pharmacological DP receptor inhibition on facilitated psoriasis under the condition of mPGES-1 deficiency. Given that PGD2 production was increased in the skin of mPGES-1−/− mice after the induction of psoriasis, we investigated the effects of PGD2 in the IMQ-induced psoriasis pathology under the condition of mPGES-1 deficiency using antagonists specific for each DP subtype DP1 and DP2. DP1 antagonist BWA868C was administered intraperitoneally to the mPGES-1−/− mice at a dose of 1 mg/kg daily for 6 days immediately before the IMQ treatment, and the psoriasis pathology was evaluated. There was no significant difference in the skin scaling and thickening scores between mPGES-1−/− mice treated with a DP1 antagonist and mPGES-1−/− mice treated with a vehicle. The DP2 antagonist CAY10471 was admi [file 41232_2025_385_MOESM1_ESM.pdf]

Figure S1

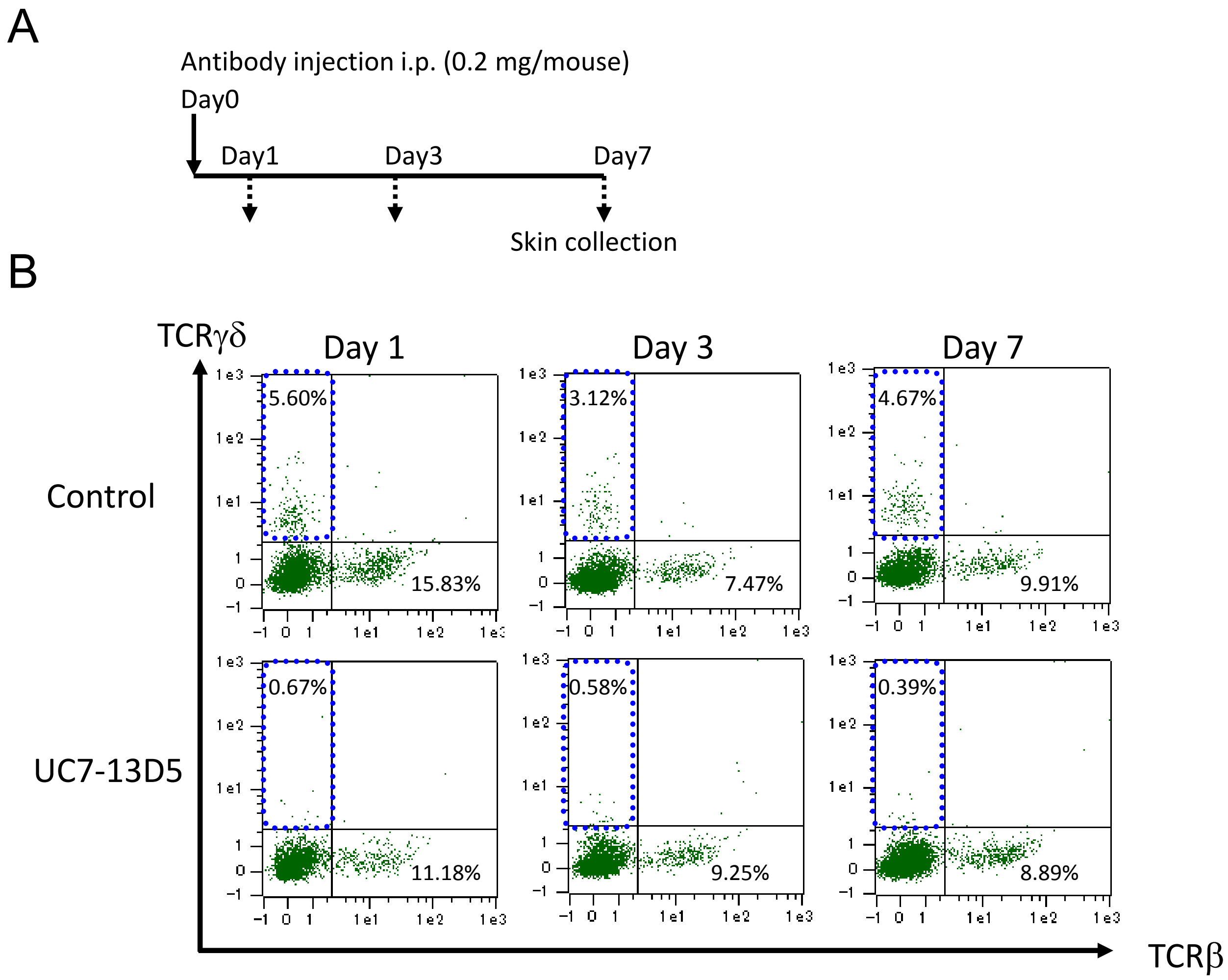

Figure S1. In vivo depletion of γδ T cells by anti-TCRγδ (clone UC7-13D5 polyclonal antibody. (A) Schematic representation of the experimental plan. Solid allows indicate timepoint (days) at which the intraperitoneal injection of the antibody were performed. (B) The efficacy of γδ T-cell depletion was confirmed through an FCM analysis of the skin samples. The administration of anti-TCRγδ antibody was successful in maintaining a significant decrease in the TCRγδ-positive γδ T cells but not TCRβ-positive αβ T cells for 7 days after the antibody administration.

Figure S2

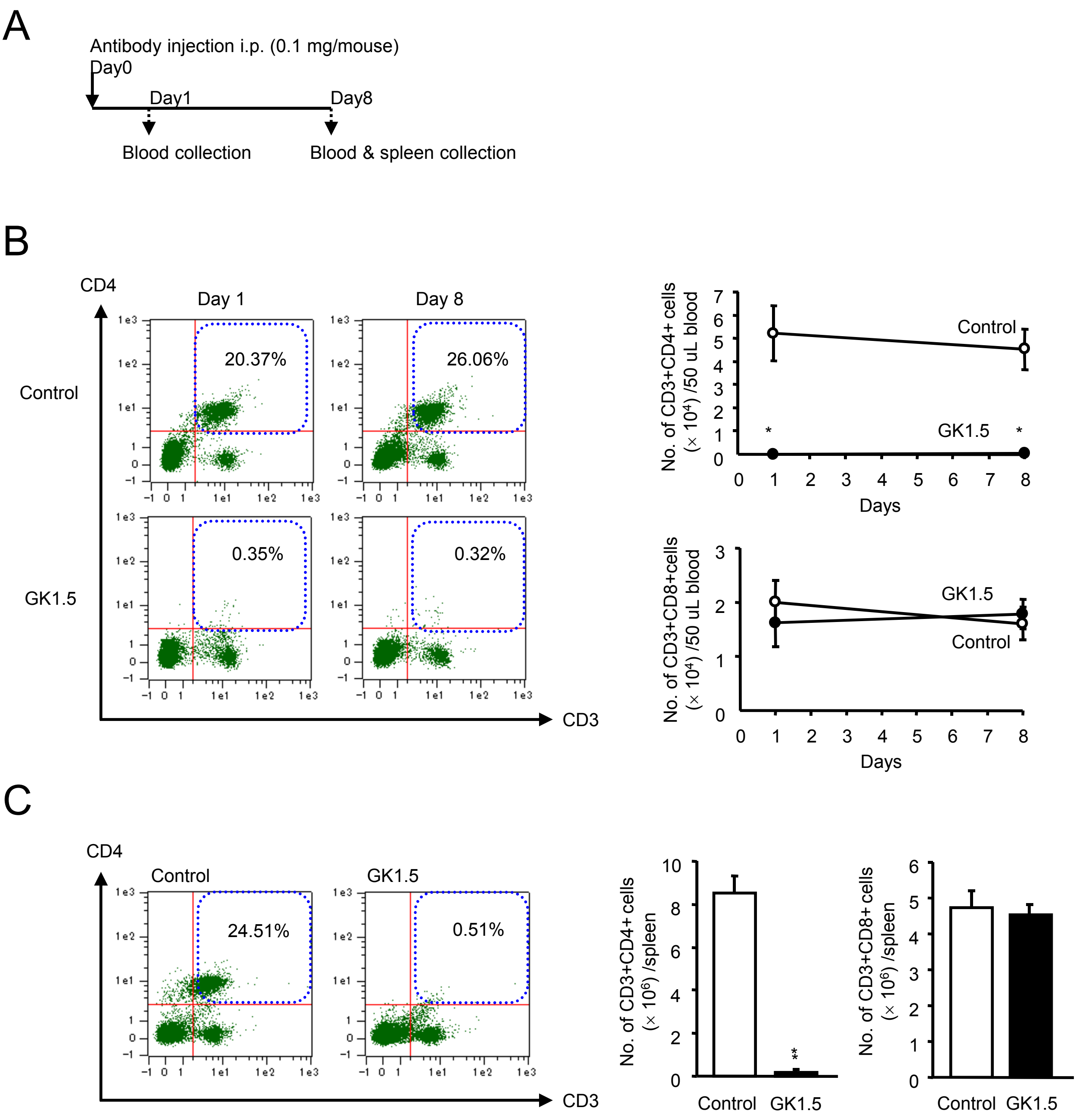

Figure S2. In vivo depletion of CD4-positive T cells by anti-CD4 (clone GK1.5) monoclonal antibody. (A) Schematic representation of the experimental plan (A). Solid allows indicate time-point (days) at which intraperitoneal injection of the antibody were performed. The efficacy of CD4-positive T cell depletion was confirmed by FCM analysis of T cell population in the peripheral blood (B) and spleen (C). FCM analysis confirmed that anti-CD4 antibody treatment effectively reduced the number of CD3+CD4+ T cells but not CD3+CD8+ T cells in the peripheral blood and spleen of the treated mice compared to the control mice. *$P < 0.05$ *vs.* control; *t* test (n=4)

Figure S3

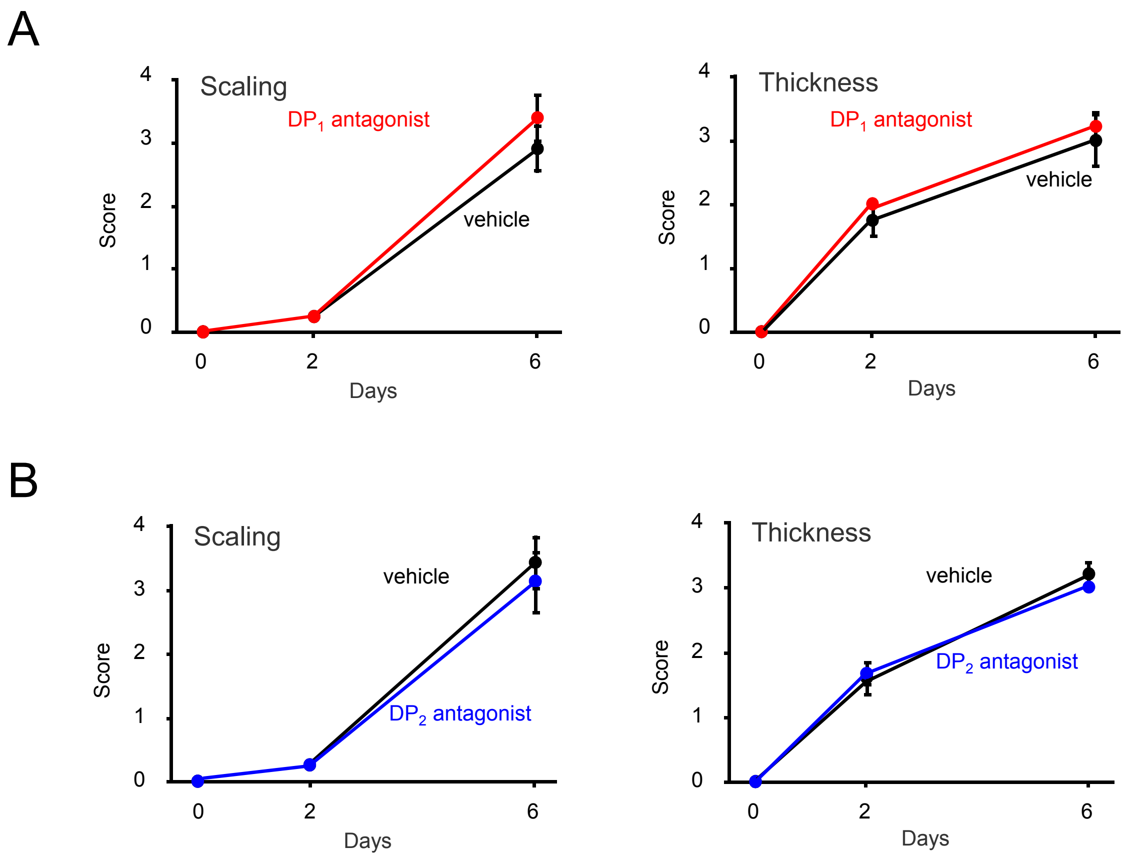

Figure S3. Effect of pharmacological DP receptor inhibition on facilitated psoriasis under the condition of mPGES-1 deficiency. Given that PGD_2 production was increased in the skin of mPGES-1$^{-/-}$ mice after the induction of psoriasis, we investigated the effects of PGD_2 in the IMQ-induced psoriasis pathology under the condition of mPGES-1 deficiency using antagonists specific for each DP subtype DP_1 and DP_2. (A) DP_1 antagonist BWA868C (Cayman) was administered intraperitoneally to the mPGES-1$^{-/-}$ mice at a dose of 1 mg/kg daily for 6 days immediately before the IMQ treatment, and the psoriasis pathology was evaluated. There was no significant difference in the skin scaling and thickening scores between mPGES-1$^{-/-}$ mice treated with a DP_1 antagonist and mPGES-1$^{-/-}$ mice treated with a vehicle (n=6–9). (B) The DP_2 antagonist CAY10471 (Cayman) was administered intraperitoneally to the mPGES-1$^{-/-}$mice at a dose of 2 mg/kg daily for 6 days immediately before the IMQ treatment. Similarly, there was no significant difference in both scores between the DP_2 antagonist and vehicle control in mPGES-1$^{-/-}$ mice (n=8–9). These results suggest that PGD_2, which is increased in the skin of psoriasis-induced mPGES-1$^{-/-}$ mice, is unlikely to exacerbate or alleviate the major symptoms of IMQ-induced psoriasis. *$P < 0.05$; ANOVA followed by Tukey's multiple comparison test.
